# Supplementary material for: Schizotrophic Sclerotinia sclerotiorum-Mediated Root and Rhizosphere Microbiome Alterations Activate Growth and Disease Resistance in Wheat
Source: Microbiol Spectr. 2023 May 22;11(3):e00981-23. doi: 10.1128/spectrum.00981-23 (PMC10269679; doi:10.1128/spectrum.00981-23)
Supplement: Supplemental file 9 — Fig. S1 and S2. Download spectrum.00981-23-s0001.pdf, PDF file, 0.2 MB [file spectrum.00981-23-s0001.pdf]

Supplemental Figures:

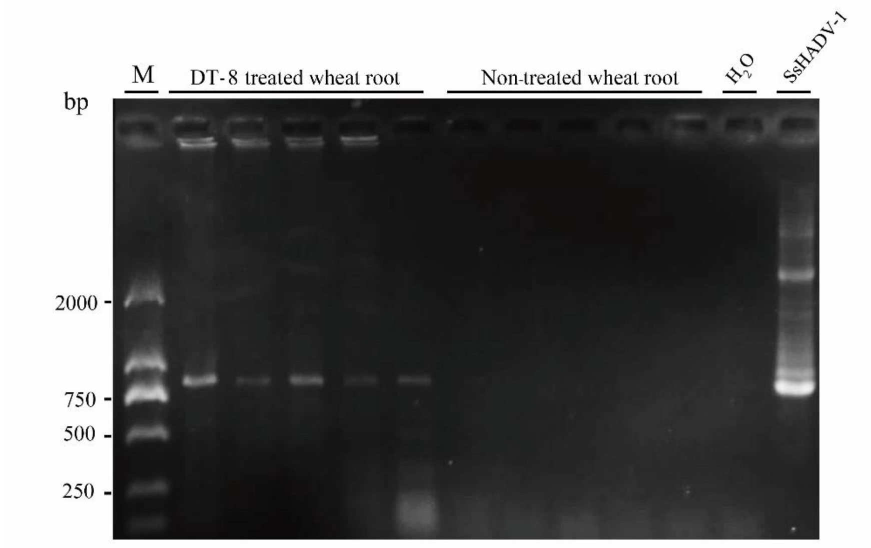

FIG S1. Detection of coat protein gene (*CP*) of SsHADV-1 in samples taken from the DT-8 treatment and control wheat roots by PCR amplification using specific primers described by Yu *et al*.

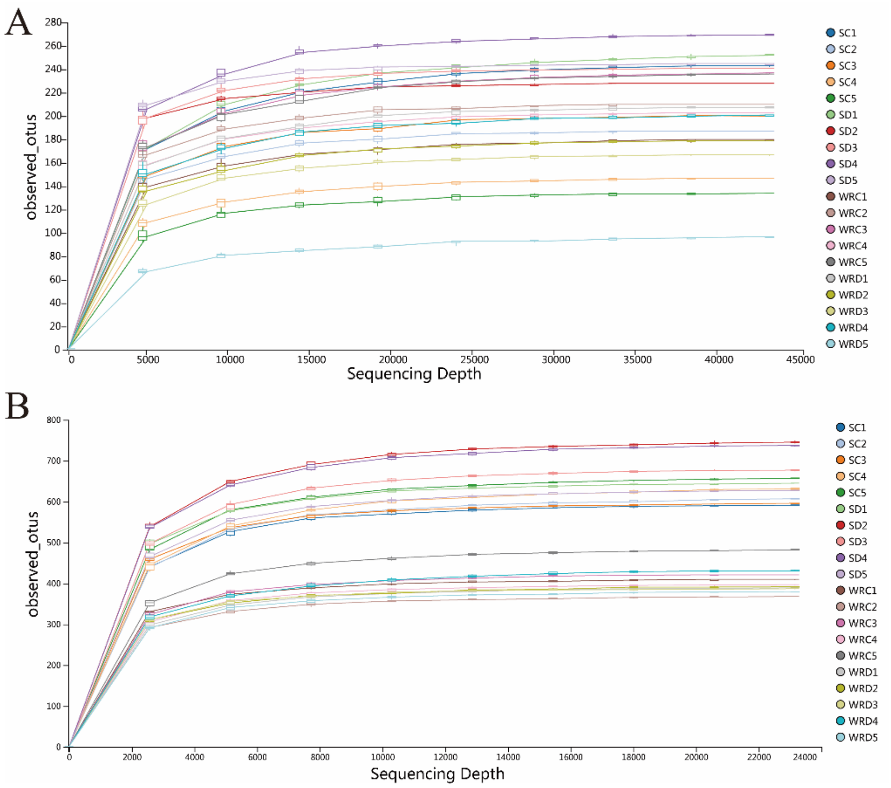

FIG S2 The rarefaction curves of ITS sequencing samples (A) and 16S rRNA sequencing samples (B).
